# Fluorescent Covalent Organic Frameworks: A Promising Material Platform for Explosive Sensing

**DOI:** 10.3389/fchem.2022.943813

**Published:** 2022-07-15

**Authors:** Yuhang Qian, Jiani Li, Mingyang Ji, Jundan Li, Dongge Ma, Anan Liu, Yubao Zhao, Chun Yang

**Affiliations:** ^1^ College of Chemistry and Materials Engineering, Beijing Technology and Business University, Beijing, China; ^2^ Basic Experimental Centre for Natural Science, University of Science and Technology Beijing, Beijing, China; ^3^ Institute of Environmental Research at Greater Bay, Guangzhou University, Guangzhou, China; ^4^ College of Life Sciences and Chemistry, Hunan University of Technology, Zhuzhou, China

**Keywords:** covalent organic frameworks, fluorescence, chemical sensing, fluorescence mechanisms, explosive sensing

## Abstract

Covalent organic frameworks (COFs) are a novel class of porous crystalline organic materials with organic small molecule units connected by strong covalent bonds and extending in two- or three-dimension in an ordered mode. The tunability, porosity, and crystallinity have endowed covalent organic frameworks the capability of multi-faceted functionality. Introduction of fluorophores into their backbones or side-chains creates emissive covalent organic frameworks. Compared with common fluorescent organic solid materials, COFs possess several intrinsic advantages being as a type of irreplaceable fluorescence materials mainly because its highly developed pore structures can accommodate various types of guest analytes by specific or non-specific chemical bonding and non-bonding interaction. Developments in fluorescent COFs have provided opportunities to enhance sensing performance. Moreover, due to its inherent rigidified structures and fixed conformations, the intramolecular rotation, vibration, and motion occurred in common organic small molecules, and organic solid systems can be greatly inhibited. This inhibition decreases the decay of excited-state energy as heat and blocks the non-radiative quenching channel. Thus, fluorescent COFs can be designed, synthesized, and precisely tuned to exhibit optimal luminescence properties in comparison with common homogeneous dissolved organic small molecule dyes and can even compete with the currently mainstream organic solid semiconductor-based luminescence materials. This mini-review discusses the major design principle and the state-of-the-art paragon examples of fluorescent COFs and their typical applications in the detection and monitoring of some key explosive chemicals by fluorescence analysis. The challenges and the future direction of fluorescent COFs are also covered in detail in the concluding section.

## Introduction

Fluorescent sensor materials are important in various aspects of human life such as the recognition and detection of explosives, toxic heavy-metal ions, hazardous anions, VOCs (volatile organic compounds), POPs (persistent organic pollutants), antibiotics, agrochemicals, and pharmaceuticals (Basabe-Desmonts et al., 2007). In addition, fluorescent sensors also play pivotal roles in bio-sensing, bio-imaging, and therapeutics (Ashton et al., 2015). Currently, the main fluorescent sensing materials can be divided into three main categories: inorganic (Bonacchi et al., 2011), organic (Wang et al., 2022), and inorganic–organic hybrid materials (Lustig et al., 2017). Compared with the inorganic and hybrid luminescent materials such as *f-*block rare-earth lanthanide complexes and nanoparticles and *d-*block transition-metal complexes and metal–organic frameworks (MOFs), pure organic fluorescent materials have exhibited their multiple advantages such as low toxicity, earth-abundance, excellent metabolism kinetics property for *in vivo* applications, low density, and malleability for wearable optoelectronics (Crowe et al., 2016). However, due to their common two-dimensional layered π–π stacking structures, significant aggregation-caused quenching (ACQ) effects often occurred in these organic solid materials. Moreover, the ultrafast excited-state non-radiative decay processes via the intramolecular motion and the intermolecular electron transfer or the energy transfer channel also accounted for their weak or non-fluorescence. Thus, it still remains a great challenge to develop highly fluorescent pure organic materials to overcome these two key challenges (Skorjanc et al., 2021).

In 2001, with the milestone discovery of aggregation-induced emission (AIE) by Tang’s group, pure organic emissive materials have been continuously burgeoning and advanced into a new era (Mei et al., 2014). Various AIEgen molecules {tetraphenylethylene (TPE), hexaphenylsilole, hexaphenylbenzene, 9,10-di[(E)-styryl]anthracene, pentaarylpyrrole, and tetraaryl-substituted 1,3-butadienes, etc} were designed and synthesized, and they exhibited strong emissive property in either aggregate or solid state. Their AIE and solid-state emissive properties originated from the restriction of intramolecular rotation (RIR) and vibration (RIV) (Li et al., 2015). Although these organic solids can be applied as fluorescent or even phosphorescent materials for numbers of applications, their lack of pre-defined and predictable stacking modes influenced the understanding of the underlying mechanism for their fluorescence because the key geometry, configuration, and conformation of these organic solid-materials mostly in non-crystalline amorphous forms are unclear.

Covalent organic frameworks (COFs), being considered the next-generation star materials for multifunctional applications, have aroused considerable research focuses for decades since its first discovery in 2005 (Côte et al., 2005). COFs are a kind of porous crystalline polymers, which are consisted of sole organic compounds and connected with covalent bonds. Unlike other porous organic polymers, COFs are crystalline since its synthetic chemistry involves thermodynamically controlled reversible chemical reactions, other than kinetically controlled irreversible reactions, which generate non-crystalline amorphous materials (Diercks and Yaghi, 2017). The synthesis of COFs usually takes several days to reach a reversible dynamic chemical equilibrium, during which the so-called “proof-reading” and “error-checking” processes occurred, which finally generate the most stable crystalline state of COF materials with the lowest energy (Ding and Wang, 2013). Compared with their metal–organic framework (MOF) counterparts, COFs possess good chemical robustness because MOFs are interwoven by weak metal–ligand coordinative bonds, whereas COFs are constructed by much stronger covalent bonds. Although the first several boronate-based COFs were unstable in humid air or aqueous environment due to the boron atom, they can facilely undergo nucleophilic attacks by H_2_O (El-Kaderi et al., 2007). In 2008, imine- and hydrazone-linked COFs marked the adventure of a water-stable COF era (Han et al., 2008). These robust COFs and the following discovered super-stable ketoenamine (Chandra et al., 2013), and sp^2^-conjugated C=C (Jin et al., 2017) COFs made the realistic applications of COFs feasible. Apart from their robustness, being consisted of only non-metal light elements, such as C, H, O, N, B, F, S, and Si, COFs usually possess very low density. Choosing diversified building blocks, various reticular linking chemistries and the plethora of post-synthetic modifications can render COFs plentiful possibility for various applications, ranging from optoelectronics, energy storage, mass transport, adsorption, separation, and catalysis to sensing applications (Feng et al., 2012).

COFs applied in the field of fluorescence will be discussed in detail in the following sections. First, this kind of highly porous materials possessing abundant interior pores can be effectively designed to selectively recognize the specific analytes and initiate responding processes by either fluorescence enhancing or quenching. Second, the controllable formation of appropriate covalent bonds in the two-dimensional plane and three-dimensional space also enhances the specific intermolecular interaction between analytes and COFs. Guided by these two major conclusions, we continue our discussion on the main examples of COF fluorescence materials applied in the field of explosive analysis. We divide our following discussion into three parts including fluorescence mechanisms of COFs, explosive sensing, and conclusion. In addition, we provide the current main challenges and the future developing directions of COFs for the fluorescence sensing of explosive analysis. We believe that this mini-review can help to provide novel insights and understandings to further design and synthesize more efficient fluorescent COF sensors.

## Fluorescence Mechanisms of Covalent Organic Frameworks

Due to the significant π–π stacking interaction between two-dimensional COF neighboring layers, even if highly conjugated and rigid organic fluorophores are introduced into COF skeletons or side-chains, two-dimensional COFs usually exhibit weak or non-fluorescence due to the strong interlayer π–π stacking interaction inducing typical aggregation-caused quenching (ACQ) effects by greatly promoting the coupling effect to quench the excited-state energy *via* heat-release routes (Dalapati et al., 2013). Although three-dimensional COFs commonly do not aggregate to induce fluorescence quenching, there are still multiple obstacles to realize the fluorescent three-dimensional COFs (Lin et al., 2016). Both the relative rarity of suitable fluorescent three-dimensional COF building blocks and the considerable difficulties in preparing long-range ordered organic polymers with crystallinity and porosity have been rendered as a challenging task to realize emissive fluorescent 3D COFs. Since the first milestone report on fluorescent COFs reported by Jiang’s group in 2008 (Wan et al., 2008), there appeared dozens of fluorescent two-dimensional and three-dimensional COFs capable for chemical sensing applications (Liu et al., 2019). Thus, it is valuable to unveil the underlying mechanism of how these fluorescent COFs realize fluorescence emission.

According to the features of fluorophore monomer units in COFs, emissive COFs are divided into two major kinds. The first family incorporates the planar ACQ π-conjugated building blocks, especially pyrene derivatives (Wan et al., 2009). Although they are constructed by ACQ monomers, the formation of COF reticular structures limited the bond free-rotation, which is a key factor inducing the ACQ process. The rigidified chemical structures greatly lock-off the bond motion, which blocked the non-radiative path, thus promoting the fluorescence. The early discovered fluorescence COFs usually belonged to this family. Although two-dimensional fluorescent COFs can be constructed *via* confining the fluorophores into the rigid reticular structures, the fluorescence intensity and the photoluminescence quantum yield (PLQY) are still far from expectation. Inspired by the previous success of AIE and solid-state emission of organic solids formed from AIEgens, which can effectively inhibit the thermal energy dissipation routes of RIR or RIV in aggregate states, some scientists borrowed this concept to the COF research field. Also, there appeared two famous fluorescent COFs, namely, TPE-Ph-COF (Dalapati et al., 2016) and 3D-TPE-COF (Ding et al., 2018). These two COFs all shared the same AIEgen-TPE and exhibited excellent performance in fluorescence intensity and PLQY. TPE-Ph-COF has been retaining the highest PLQY record since 2016 until now, and this COF sensor detected traces of toxic gas NH_3_ in μM low level (Dalapati et al., 2016). Both exhibited enhancing fluorescence compared with the amorphous polymers and crystalline model compounds. Due to the simplicity in synthesis and the satisfactory fluorescence performance, TPE units are currently the most common AIEgen incorporated into COF structures. Apart from two-dimensional pyrene-based and TPE-based fluorescent COFs, 3D-TPE-COF is the first fluorescent three-dimensional COFs, and it acted as effective sensing materials to quantitatively analyze the trace amount of picric acid. Three-dimensional COFs provide another solution to tackle the dilemma between the requirement of π-conjugated planar fluorophore monomers and the severeπ–π stacking quenching. Because it does not necessitate the co-planar conformations as in two-dimensional COFs, three-dimensional COFs effectively exploit the potential of π-conjugated fluorophore monomers by threading them in a non-coplanar 3D-extending manner. The concrete solution is to use T_d_ knot to break the conjugation and to enhance the non-coplanarity, whereas the linker TPE-CHO units are accountable for strong fluorescence emission (Ding et al., 2018).

## Covalent Organic Frameworks as Explosive Sensors

As porous polymeric materials, COF materials function as a powerful host to accommodate the analyte guest molecules by their highly developed one-dimensional channel and size-controllable interior pores. Moreover, by precisely fine-tuning the electronic and steric factors of monomers, the linking reticular chemistry and the stacking modes, specific interactions between analyte molecules trapped inside the pores, and COF recognition and fluorescence units can be well established. By the selective binding of analyte guest molecules, the intrinsic photo-induced electron-transfer (PET), Förster resonance energy transfer (FRET), Dexter energy transfer, and inner-filter effects (IFE) can occur to selectively turn on or block off the fluorescence signals. Otherwise, when the COF fluorescent monomer coordinated with the analyte, the structural changes such as deprotonation, protonation, and tautomerization will trigger the fluorescent change of COF materials. Thus, the linear relationship can be established based on the fluorescence intensity change with the concentration of the analyte. To realize meaningful COF chemo-sensing applications, enhancing the selectivity with the specific guest molecules and increasing the sensitivity by responding to more diluted analytes are the two major developing directions.

Nitrobenzene explosives are used as important chemical compounds in mines, military, and safety administration. But they are also severe pollutants in the natural aqueous environment. Moreover, they are used as explosives by terrorists, causing panic and threatening the lives of citizens. The rapid and accurate detection of nitrobenzene and other explosives now becomes a particularly urgent issue (Germain and Knapp, 2009). Due to their adjustable structure, developed pore structures, and satisfactory stability under aqueous, heating, acidic, or basic conditions, COF materials have attracted the attention as a promising star material platform for explosive sensing, especially nitrobenzene analogs.

As mentioned previously, most two-dimensional COFs are non-fluorescent or weakly fluorescent even by incorporating fluorophore monomer units due to their severe interlayer π–π-stacking-induced fluorescence quenching. This may also be due to the much greater kinetics rate constant of thermal deactivation channel in comparison with relatively slow fluorescence kinetics. Moreover, two-dimensional COFs exhibited a low fluorescence intensity compared with their organic small molecule partner mainly because of the higher non-radiative deactivation rate constant due to the strong interlayer coupling. Using a strong luminescent monomer such as pyrene can re-open COF fluorescence channels.

Jiang et al. synthesized the first fluorescent COF sensor for explosives by condensing hydrazine with 1,3,6,8-tetra-(4-formylphenyl)pyrene under the solvothermal condition (Dalapati et al., 2013). The as-synthesized COF possesses not only strong fluorescence but also satisfactory stability and crystallinity. This azine-linked COF selectively detected TNP(1,3,5-trinitrophenol) explosive to 70 ppm concentration even in the presence of other nitrobenzene interferences. The high sensitivity and selectivity originate from the synergistic interaction between pyrene vertices, azine edges, and the whole π-conjugated skeleton.

**FIGURE 1:**
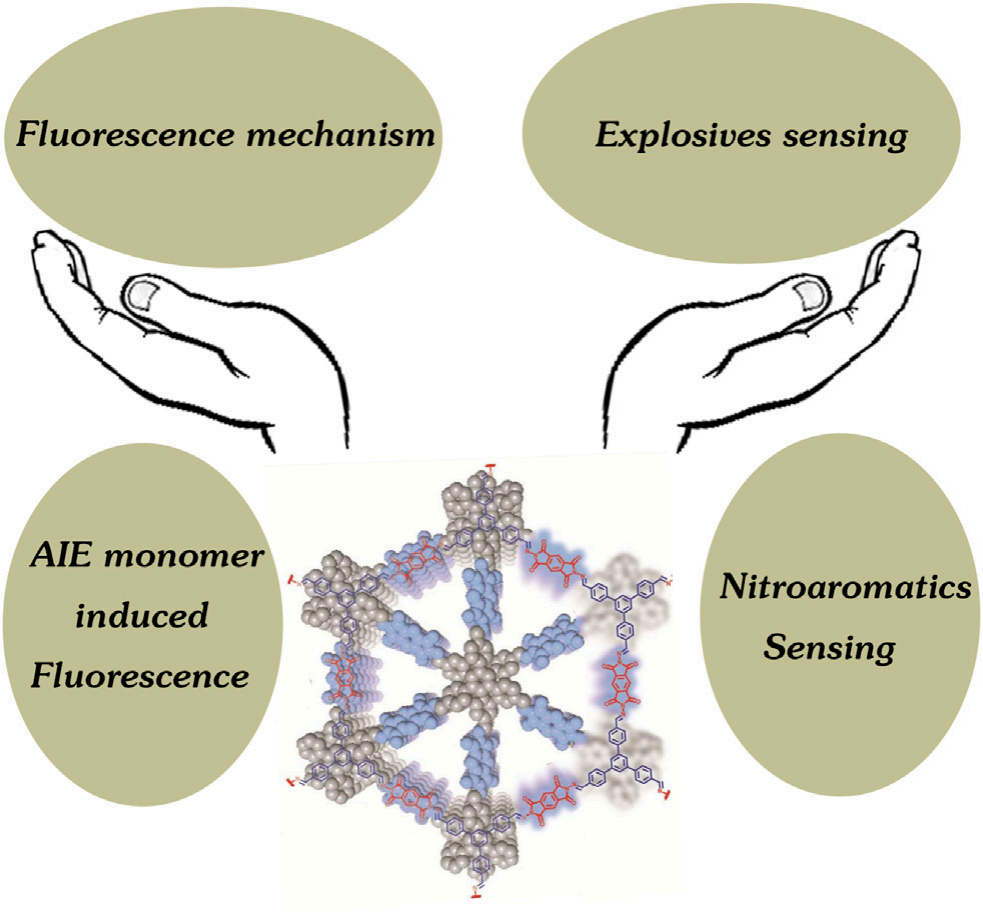
Schematic presentation of COF fluorescence mechanism and explosive sensing.

**FIGURE 2:**
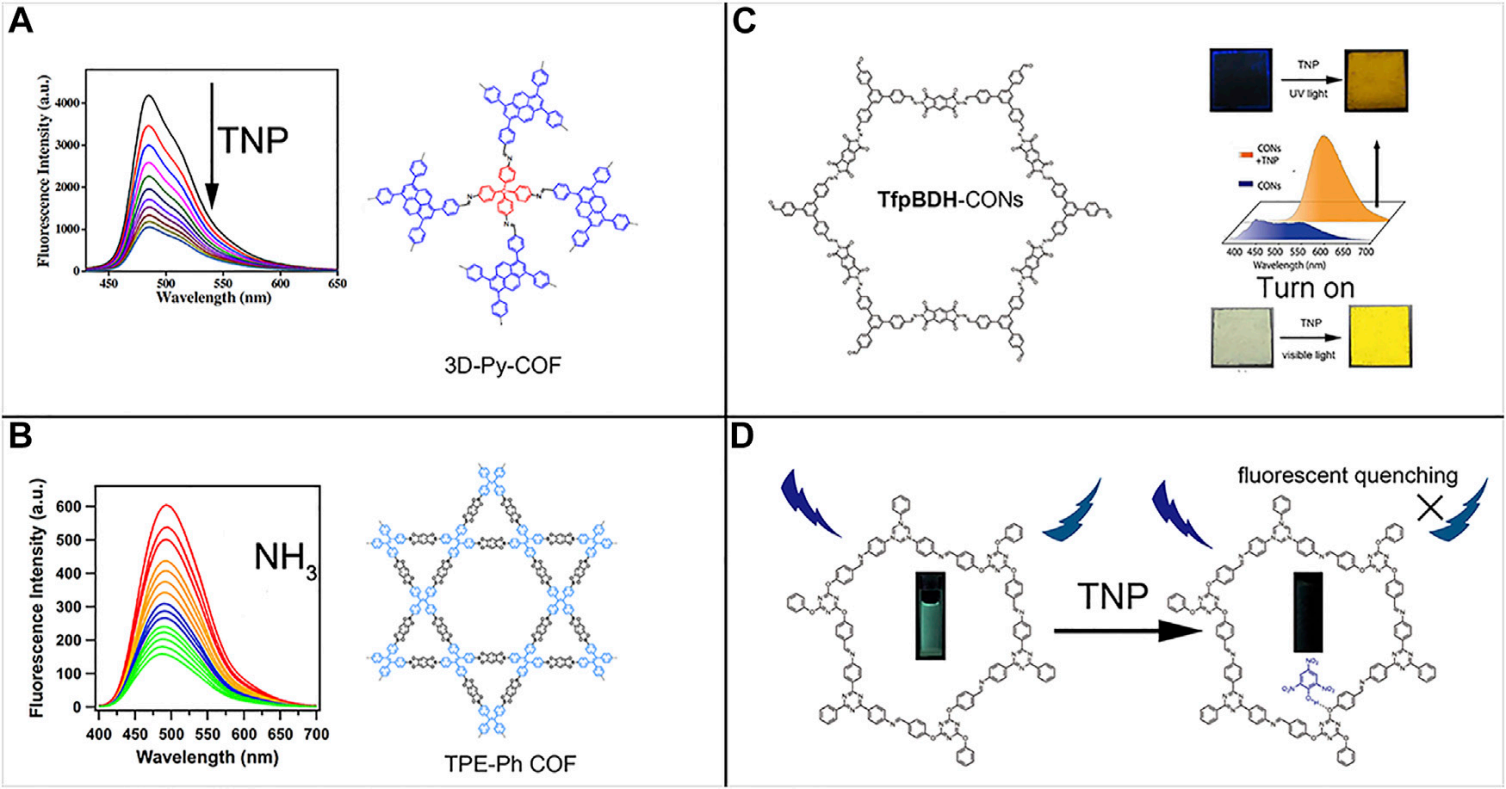
**(A)** Three-dimensional non-conjugation-induced fluorescent 3D-Py-COF (Lin et al., 2016). **(B)** AIEgen-monomer-induced fluorescent two-dimensional TPE-Ph-COF (Dalapati et al., 2016). **(C)** TfpBDH-CONs solid-state sensing to TNP under UV (365 nm) and visible light in a turn-on mode (Das et al., 2015). **(D)** DTZ-COF turn-off fluorescence response to TNP (Li et al., 2019). Adapted with the permission from the American Chemical Society and the Royal Society of Chemistry.

Apart from azine-COFs, Banerjee et al. exhibited that imide TfpBDH COF and CON (covalent organic nanoplate) effectively detected TNP with a turn-on mode (Das et al., 2015) (see Figure 1). Moreover, when deposited on a strip paper, the mM-level TNP can be recognized even by the naked eyes. This TNP probe is sensitive and selective with the lowest detection limit (LOD) as 10^−5^ M, and it functioned without pronounced response in the presence of other nitroaromatics such as TNT, DNT, DNP, NT, and NP. The good sensing performance relates with the aggregation state of CON, which on the one hand accommodated more available electrons to participate in the electron-transfer and energy-transfer process with TNP analyte. On the other hand, the few-layered CON greatly decreased the interlayer π–π stacking regenerating the fluorescence channel Figure 2.

Imine-linkages are the most common COF motifs. However, due to the relatively flexible single bonds near the imine bond, they usually suffer from total or partial fluorescence quenching. This property considerably limited their uses as chemo-sensors. Loh et al. utilized the morphology engineering tactic to increase the imine-COF fluorescence intensity (Gao et al., 2018). They fabricated a two-dimensional Py-TPE COF with spherical particle morphology. They considered that the imine bond rotation was restrained because of the curvature-induced strain. Moreover, the introduction of a non-planar AIEgen TPE group effectively mitigated π–π stacking effects. As calculated from the PXRD (001) lattice signal, the interlayer distance is approximately 4.0 Å, indicating a weak π–π stacking interaction. The COF PLQY was as high as 21.1% in CH_3_CN dispersion, which is the highest recorded PLQY for the imine-bonded COFs. The Py-TPE COF acted as a selective and sensitive turn-off sensor for the detection of picric acid with the ppm level LOD. Other nitroarene interferences such as TNT, DNP, DNT, NT, and NP cannot generate remarkable fluorescence quenching. This indicated that the proper design and engineering of COF morphology can also provide the alternative effective strategy for nitrobenzene explosive sensing.

Apart from the rigid pyrene and TPE-like AIEgen monomers as two-dimensional fluorescent COF building blocks, the flexible TPOT-CHO and TPT-NH_2_ formed a two-dimensional DTZ-COF, which exhibited high crystallinity with intense XRD peaks at 2θ < 10^o^ and remained highly porous with a 1276 m^2^/g BET specific area (Li et al., 2019). The π–π stacking was attenuated in this DTZ-COF due to the destruction of co-planar configuration by TPOT flexible knots. It acted as an efficient fluorescent turn-off probe for TNP. The sensitivity on picric acid (TNP) realized sub-ppm LOD (81.8 ppb). The TNP phenolic group acted as a hydrogen bond donor, creating a hydrogen bond with the imine C=N forming ground-state complex and effectively quenching the fluorescence. On the other hand, the conduction band (CB) electrons of COFs possess higher energy level than the LUMO of TNP, and the excited-state electrons of DTZ-COF can transfer to TNP LUMO, leading to fluorescence quenching by PET (photo-induced electron transfer).

Das and Mandal, (2018) realized TNP analysis with a 68 ppb LOD by incorporating a strong electron-deficient naphthalene diamine (NDA) moiety into a two-dimensional COF skeleton. The PET is the most probable sensing mechanism. The suitable energy band alignment guaranteed the high selectivity. In addition, the emission spectra of COF overlapped largely with TNP absorption. Thus, the FRET mechanism may also contribute to fluorescence quenching. Also, the 68 ppb detection limit of TNP is the low record among the previously reported TNP COF fluorescent sensors.

Combining AIEgen TPE-NH_2_ and conjugated planar anthracene dialdehyde monomer, Faheem et al. (2019) synthesized a novel rhombic dual-fluorescence covalent organic framework (DL-COF). It exhibited excellent sensitivity and selectivity toward nitroaromatics. Its LOD for nitroarenes is at ppb level which rivals all the previous COF- and MOF-based nitroaromatic fluorescence sensors. Moreover, from their Stern–Volmer plot, the quenching coefficient K_SV_ is about 10^6^ M^−1^, which is several orders larger than that of the other COF and MOF nitroaromatic sensor. The authors attributed this quenching to two key factors. On the one hand, the formation of non-emissive complex at the ground-state, i.e., the static quenching contributed to the fluorescence quenching upon addition of nitroaromatics. On the other hand, the dynamic quenching by electron/energy transfer is the main factor for the effective fluorescence quenching. By the synergistic utilization of the static and dynamic quenching, DL-COF demonstrated its power to detect the trace amount of nitroaromatics. However, the inability to recognize different nitroaromatic compounds is a flaw, and the selectivity can be further improved. Furthermore, effective turn-on fluorescence sensing is also desired to be developed for more sensitive detection without special expensive spectroscopy equipment.

Different from the common two-dimensional COF fluorescent nitroaromatic sensors, Wang et al. chose an alternative strategy to design and synthesize 3D-Py-COF (Lin et al., 2016). The as-synthesized three-dimensional COF adopted a novel interpenetrated pts topology. Due to the relief of the interlayer π–π stacking interaction and the incorporation of photoelectronic active pyrene units, this 3D-Py-COF exhibited strong blue-greenish fluorescence emission at 484 nm upon excitation at 408 nm. Moreover, this three-dimensional fluorescent COF can act as an effective picric acid sensor (PA) with a quenching detection mode upon the addition of PA from 0 to 20 ppm. This was the first three-dimensional fluorescent COF and can be used as nitroarene sensors.

Not only nitroarene explosives can be detected by various two-dimensional and three-dimensional COF materials, but more destructive triacetone trioxide (TATP) explosives, which lack the typical electron-accepting nitroarene motif, can also be efficiently detected by a novel 2D-conjugated COF. Perepichka et al. constructed the ketonenamine COF by a Michael addition–elimination cascade reaction between β-ketoenols and aromatic amines (Rao et al., 2017). The as-synthesized COF possessed considerable stability in comparison with boronate- and imine-linkage COFs. The keto-enamine COF exhibited better π–electron delocalization than the previously reported imine COFs. Moreover, this COF functions as an effective fluorescent quenching probe toward both nitroarene and TATP peroxide explosives. The authors attributed the fluorescence quenching of TATP to the oxidation of COF by TATP, which was evidenced by the change of COF color and the simultaneous red-shifting in UV-Vis-NIR absorption spectrum. The authors provided an alternative COFs linking chemistry, i.e., Michael-addition reaction, and this new dynamic chemical reaction has generated COFs with more electron delocalization and realized the unprecedented challenging TATP peroxide explosive detection task.

Apart from the experimental results on the application of fluorescent COFs for explosive detection, Hussain et al. (2019) also conducted theoretical calculations to elucidate the mechanisms for the common COF fluorescence quenching phenomena toward nitroarene explosives (Hussain et al., 2019). Taking LCOF-BTT1 as an example, they discovered that the hydrogen bonding played a pivotal role for the fluorescence quenching. Applying the density functional theory (DFT) and time-dependent density-functional theory (TDDFT) to simulate the ground states and the excited states of both the LCOF and the nitroarene-binding COF complex, the results indicated that the complex possesses stronger hydrogen bonds than the pristine LCOF. Also, the stronger hydrogen bonds produced much faster internal conversion rates. Accelerated internal conversion contributed to the lower fluorescence intensity. Thus, the fluorescence quenching of the luminescent COFs upon the addition of nitroarene explosives can be partly attributed to hydrogen-bonding-enhanced non-radiative internal conversion processes.

## Discussion

As a class of novel materials, COFs have been developed rapidly for almost 2 decades since 2005 in Yaghi’s inaugurating work (Côte et al., 2005). The COF research field has changed from the infantile phase to be more and more mature and specific. As in the field of fluorescence sensing of explosives, COF materials have already accumulated many successful examples. However, most of them can only realize this task by incorporating TPE-like AIEgens as knots or linkers. It is highly desired to design and prepare other types of building blocks for fluorescent COFs. The second fact is that the recent examples mainly concentrated on the nitrobenzene explosives, where other typical explosives such as black powder, Hg(CNO)_2_, RDX, and TATP are less concerned. Much more focuses can be encouraged to highlight these explosives, and more efforts can be devoted on developing more general and broad-spectrum sensors for more types of explosives. Another direction for COF fluorescence explosive detection is to develop more fluorescence turn-on sensors and naked-eye detection. Higher intrinsic fluorescence intensity and PLQY should be pursued. Possessing satisfactory sensitivity, selectivity, and stability, we believe that the future of fluorescence COFs will be brighter if we continue to involve efforts on this significant research area.
